# Quality care process metrics (QCP-Ms) in nursing and midwifery care processes: a rapid realist review (RRR) protocol

**DOI:** 10.12688/hrbopenres.13120.2

**Published:** 2021-01-28

**Authors:** Laserina O'Connor, Alice Coffey, Veronica Lambert, Mary Casey, Martin McNamara, Sean Paul Teeling, Jane O'Doherty, Marlize Barnard, Yvonne Corcoran, Carmel Davies, Owen Doody, Timothy Frawley, Denise O'Brien, Catherine Redmond, Rita Smith, Suja Somanadhan, Maria Noonan, Carmel Bradshaw, Dympna Tuohy, Anne Gallen

**Affiliations:** 1 School of Nursing Midwifery & Health Systems, University College Dublin, Dublin, Ireland; 2 Department of Nursing and Midwifery, University of Limerick, Limerick, Ireland; 3 School of Nursing, Psychotherapy and Community Health, Dublin City University, Dublin, Ireland; 4 UCD Health Sciences Centre, University College Dublin, Dublin, Ireland; 5 Director of Nursing and Midwifery Planning and Development Unit (NMPDU), Health Service Executive North West, Donegal, Ireland

**Keywords:** rapid realist review, quality care metrics, care processes, quality indicators

## Abstract

**Background: **In 2018, the Office of the Nursing and Midwifery Services Director (ONMSD) completed phase one of work which culminated in the development and launch of seven research reports with defined suites of quality care process metrics (QCP-Ms) and respective indicators for the practice areas – acute care, midwifery, children’s, public health nursing, older persons, mental health and intellectual disability nursing in Ireland. This paper presents a rapid realist review protocol that will systematically review the literature that examines QCP-Ms in practice; what worked, or did not work for whom, in what contexts, to what extent, how and why?

**Methods** **:** The review will explore if there are benefits of using the QCP-Ms and what are the contexts in which these mechanisms are triggered. The essence of this rapid realist review is to ascertain how a change in context generates a particular mechanism that produces specific outcomes. A number of steps will occur including locating existing theories on implementation of quality care metrics, searching the evidence, selecting relevant documents, data extraction, validation of findings, synthesising and refining programme theory. This strategy may help to describe potential consequences resulting from changes in context and their interactions with mechanisms. Initial theories will be refined throughout the process by the local reference panel, comprised of eight key intervention stakeholders, knowledge users such as healthcare professionals and an expert panel. Ethical approval is not required for this rapid realist review.

**Conclusion: **It is anticipated that the final programme theory will help to explain how QCP-Ms work in practice; for whom, why and in what circumstances. Findings of this review could help to give insights into the use of a rapid realist review as a framework and how nursing and midwifery QCP-Ms have been implemented previously.

## Introduction

The delivery of care to any patient/resident/woman/child and family should be of high quality, consistent, safe and patient-centred. Nurses and midwives are at the centre of care delivery, delivering high quality and safe care to patients and their families ^1^. The World Health Organisation’s (WHO) global strategic directions (2016–2020) provides a framework for the WHO and key stakeholders to create, implement and evaluate nursing and midwifery accomplishments to ensure acceptable, good quality, and safe nursing and midwifery interventions ^2^. High quality care delivery is important both in Ireland and internationally and quality measures such as metrics play a part in helping to standardise care and ensure consistency in quality ^3^. In many organisations, there is a wealth of data but often, there is no way to “collect, analyse and interpret data that will track the quality of care delivery” ^1^. The WHO (2006), defined high quality of care as the extent to which health care services provided to individuals and patient populations improve desired health outcomes. In order to achieve this, health care must be safe, effective, timely, efficient, equitable and people-centred ^4^. 

In 2011, Nursing and Midwifery Planning & Development Units (NMPDU) developed and implemented quality care-metrics (QCP-Ms) in over 100 practice areas across the Republic of Ireland ^5^. These QCP-Ms were endorsed by the Office of the Nursing & Midwifery Services Director (ONMSD) Health Service Executive ^1^. QCP-Ms are “a measure of the nursing and midwifery clinical care processes, in healthcare settings in Ireland, aligned to evidenced-based standards and agreed through consensus” ^6^. It is important to measure the degree to which nurses and midwives adhere to fundamental care processes to access and assure the quality of care delivered to patients and clients ^6^. Quality measures are frequently classified into three types: structure, process and outcomes ^7^. Structure reflect factors such as the availability of staff and facilities, process considers whether care interventions adhered to best practice guidance, and outcomes consider the changes because of care delivered. Further, nursing metrics are agreed standards and benchmarks ^8^. According to the HSE, QCP-Ms are sensitive to the influence of nurses and midwives appropriate for all care settings, aligned to evidence-based standards and agreed through national consensus. Nursing Quality Care Process Metrics provide the framework to identify gaps in care delivery, enabling action planning for quality improvement and provide a mechanism by which care providers can be accountable for the quality of their care delivery ^5^. 

In June 2018, ONMSD completed phase one of work, which included a systematic review and a modified four-round Delphi study. Phase one identified important aspects of nursing and midwifery interventions/care processes that should be measured (Nursing & Midwifery-Sensitive Process Metrics) ^1^. Implementing changes in practice are complex ^9^ and some changes are more likely to be implemented than others ^10^. QCP-Ms phase one culminated in the development and launch of seven research reports with defined suites of metrics for the following clinical practice areas – acute care, midwifery, children’s, public health nursing, older persons, mental health and intellectual disability nursing ^11^. A total of 91 metrics were launched (acute care – 11 metrics; midwifery – 18 metrics; children’s – eight metrics; older person’s care – 19 metrics; public health nursing – 14 metrics; mental health nursing – nine metrics; and intellectual disability nursing – 12 metrics) ^11^. A national procedural guideline document for each suite of QCP-M’s was also developed to guide implementation, measurement and support fidelity of interpretation ^11^. The implementation of the QCP-Ms project into an individual service is structured within a framework consisting of four stages: “initiation, planning, implementation and mainstreaming” ^5^. These stages are further subdivided into 15 individual steps; from step one where an invitation is sent to each service to step 15 where the QCP-Ms have been implemented within their service and the project is monitored, reviewed and evaluated ^5^.

The rapid realist methodology aims to highlight the impact interactions have among contexts and what impact mechanisms have on intervention outcomes ^12^. The basic question of a rapid realist review (RRR) is ‘what is it about this intervention that works in this context and why’? ^13^. An RRR works on understanding what are the contexts (C), mechanisms (M), and outcomes (O) that enable or constrain the implementation of an intervention ^11^. This RRR, as part of a larger evaluation, namely phase two, will look at how in re-+lation to the intervention of quality care process metrics in practice, using rapid realist methodology to focus on how interventions work across contexts; what worked, what did not work, for whom, why and in what circumstances.

### Why a rapid realist review?

Realist review seeks to develop a common understanding of underlying factors and causative mechanisms and, according to Pawson *et al*. (2005), seeks to understand the components of the social world and stratifications of social reality. In realist inquiry there is a focus not only on ‘what works’ but on ‘what works for whom, why it works, and in what circumstances’ ^12, 14^. Realist review often involves analysis of existing data. Pawson and Tilly address the question ‘what works, for whom, in what circumstances, and how?’ within a range of interventions ^12^. The assumption is that programmes are ‘theories incarnate’ ^9^, which means when a programme is designed, it is underpinned by one or more theories about what ‘might cause change’ ^12, 15^. According to Pawson *et al.*, (2005), the iterative, flexible nature of realist reviews do not align well with protocol-driven, standardised processes common to established systematic review methods. The steps undertaken in this study will be informed by an RRR methodology, will be iterative in nature and will be revisited iteratively throughout the RRR process. 

RRR methodology was developed as a tool for applying a realist approach to a knowledge synthesis process, thereby producing a product that is useful to policy makers in responding to time-sensitive and/or emerging issues where there is limited time and resources ^13^. When undertaking an RRR, theories within the programme are explicit about how, and for whom, to what extent, and in what contexts a programme might ‘work’ ^15^. Data collected should include: “programme impacts and the processes of programme implementation, the specific aspects of programme context that might impact on programme outcomes, and how these contexts shape the specific mechanisms that might be creating change” ^16^. By testing context-mechanisms-outcomes (CMO) configurations, will enable us to understand how, why, for whom, and in what contexts the intervention, i.e., QCP-M’s are more or less likely to work and how it produces its desired and undesired outcomes ^14, 15^. Underpinned by realist evaluation methodology (12) rapid realist review (RRR) has emerged as a popular approach in health service research and is well suited to a national evaluation of implementing QCP-Ms in Nursing and Midwifery practice. Unlike a systematic review which will control context, a realist review embraces contextual complexity making it a very appropriate approach for studying healthcare implementation and quality improvement ^17^. It focuses on understanding how an intervention (i.e., QCP-Ms) works (or not) within a particular context, taking account of individual behaviours and system influences ^12, 18^. A RRR will identify the enabling and constraining dynamics influencing implementation. RRR review involves analysis and interpretation of existing data, in essence, it is the application of the realist approach to retrospective literature reviews (Pawson, 2002). RRR acknowledges that theories cannot and do not always offer explanations or predict outcomes in every context; for example, in patient safety programmes ^19^. However, RRR is an approach that suits situations where policymakers and practitioners require guidance to inform emergent decisions in practice. The ‘rapid’ review is deliberately intended to streamline evidence synthesis and provide practical outcome-based results in a short time frame. Using an expert panel, it directly engages policy makers and knowledge users in the process which ensures the review remains relevant to the practice context ^13, 17^. While this review directly informs QCP-Ms implementation in Nursing and Midwifery Practice in Ireland, it will also have transferrable learning for any other discipline or health system implementing similar improvement efforts.

### Research question

What factors enable the successful implementation of a suite of quality care process nursing/midwifery metrics across all areas in nursing and midwifery practice?

Additional sub-research questions

In nursing and midwifery quality care process metrics, what contexts and mechanisms lead to positive implementation outcome?
In nursing and midwifery quality care process metrics, what contexts and mechanisms lead to negative implementation outcomes?
What were the dominant outcome patterns in identified contexts?

## Methods

### Protocol

This study aims to conduct an RRR that involves a synthesis of the international literature (published and grey) that generates programme theories to determine a better understanding of enablers and constraining influences related to the implementation of nursing/midwifery quality care process metrics. This study will aim to unpack the mechanism of how complex interventions (QCP-Ms) work (or why they fail) in particular contexts and settings.

### Procedures

**Figure 1.**
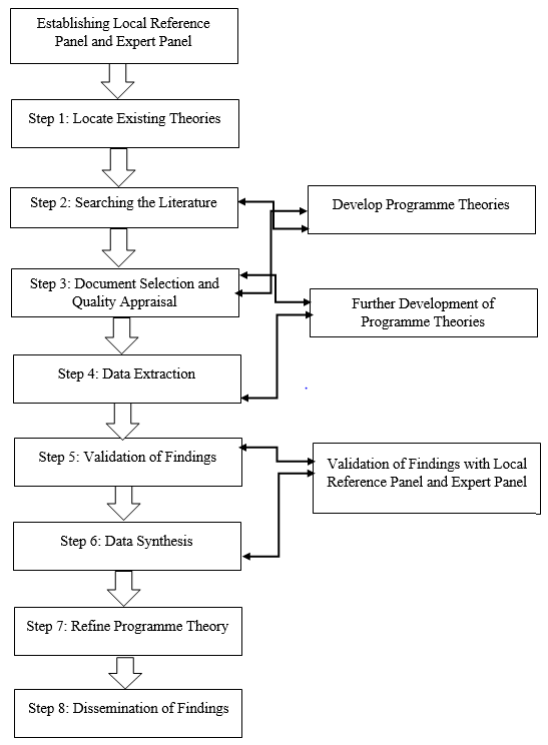
Review design.

Formal ethical approval is not required for this RRR. This RRR will be undertaken over a three-month period commencing middle of January 2021. The RRR will be informed and reported according to RAMESES (Realist And Meta-narrative Evidence Syntheses: Evolving Standards) standards ^16^. The review design is based on Weetman *et al*. (2017) study design using eight steps ^20^. The design is summarised in Figure 1. A PRISMA-P checklist has been completed and is available as an additional file (see *Extended data* ^21^).

### Step 1: Locating existing theories

Locating existing theories on what factors enable the successful implementation of a suite of QCP-Ms across nursing and midwifery practice is pertinent to step 1. We will conduct a preliminary background search in PubMed and EMBASE searching article titles, abstracts, keywords, and subject headings to guide the development of the second search strategy. Creating a good and clear search is vital to ensure the best possible and most relevant return of articles. We will use Boolean operators: AND, OR, NOT, to help define our search. Documents sourced within the scoping search will be reviewed and interrogated for theories related to what aids in the successful implementation of interventions such as QCP-Ms. The initial programme theory will be developed throughout the review process, but it will serve as a starting point for the RRR. From the findings of this scoping search and content expertise of the research team, an initial programme theory will be defined and refined throughout the RRR process. Once the initial programme theory has been developed, the searching phase will commence. 

### Step 2: Searching

We will search electronic databases including Excerpta Medica Database (EMBASE), PubMed Central, The Cumulative Index to Nursing and Allied Health Literature Complete (CINAHL Complete), APA PsycINFO, Applied Social Sciences Index and Abstracts (ASSIA) and Cochrane Database of Systematic Reviews (CDSR) (see *Extended data* ^21^) and grey literature will also be selected to include only publications within the last ten years (see *Extended data* ^21^). A PICO framework will be used to structure the key words used in the search strategy ^22^. ‘P’ in the PICO framework refers to the population, namely nurses and midwives. ‘I’ refers to an intervention, and this was the QCP-Ms. ‘C’ refers to the comparison or control group. ‘C’ can also refer to study characteristics, i.e., study design. ‘O’ refers to outcome and relates to the core research question: What factors enable the successful implementation of a suite of quality care process nursing/midwifery metrics across all areas in nursing and midwifery practice ( Table 1)?

**Table 1.** PICO search terms used in the review of the literature.

Question	PICO	Search Terms
What factors enable the successful implementation of a suite of Quality Care Process Nursing/Midwifery Metrics in nursing and midwifery practice	P I C O	‘Nurse’ OR ‘Midwife’ OR ‘nurse specialist’ OR ‘nurse practitioners’ OR ‘clinical nurse specialists’ OR ‘midwife specialist’ AND ‘quality care’ OR ‘clinical care’ ‘nursing care’ and ‘measurement’ and ‘processes’ and ‘indicators’ as separate terms AND ‘comparison groups’ OR ‘control group’ OR ‘intervention group’ OR ‘intervention groups’ AND ‘facilitators’ OR ‘enablers’ OR ‘implementation’

### Step 3: Document selection

The search strategy will be developed and agreed by the research team. Following this, the eligibility criteria will be developed. Table 2 provides an overview of the eligibility criteria for this RRR. Independent reviewers will use the online software ‘Covidence’ to import the search results and begin the title and abstract screening process ^23^. Following this, the titles and abstracts will be screened by two teams of two reviewers independently. Similarly, full text articles will be screened by two teams of two reviewers independently. ( Table 2). An arbitrator from within the research team will be appointed to discuss any discrepancies that may have emerged. If there are any discrepancies regarding which articles to include or exclude and/or why, an independent arbitrator will be consulted to make the final decision (see *Extended data* ^21^, for the PRISMA flow diagram template ^18^ that will be completed, including all numbers finalised, at the end of the RRR).

**Table 2.** Inclusion and exclusion criteria.

Criterion	Inclusion	Exclusion
**Population**	Humans	Any study population other than humans, i.e., animal studies
**Language**	Written in English	Any other language that is not in English
**Time period**	January 2010 – July 2020	Outside this time period
**Study focus**	Studies that report on the implementation and/or evaluation of the implementation of QCP-Ms or other nursing quality care measurement processes, both nationally and internationally AND Interventions including barriers and facilitators to implementation and adoption of these healthcare initiatives	Articles that do not look at QCP-Ms/ other healthcare interventions/ initiatives
**Type of study**	Peer reviewed primary studies from academic journals and grey literature e.g. reference list and institutional repositories	Non peer reviewed articles e.g. newspaper articles, opinion pieces, reviews
**Geographic** **location**	Any location – an international context	None

### Quality appraisal

There are many different critical appraisal tools outlined within the literature that enable the quality assessment of papers. These include The Crowe Critical Appraisal Tool (CCAT) ^24^ or Critical Appraisal Skills Programme (CASP) ^25^ checklists that include: Systematic Reviews, Randomised Controlled Trials, Cohort Studies, Case-Control Studies, Economic Evaluations, Diagnostic Studies, Qualitative studies and Clinical Prediction Rule ^24^. The CCAT critical appraisal tool will be utilised to ensure the highest quality papers and grey literature will be included in the review. More importantly, the CCAT is a guide to become more objective in assessing papers (personal correspondence, M. Crowe, September 24, 2020). Grey literature will not be excluded based on quality scores but will be reviewed by the local panel for inclusion or exclusion. Moreover, in terms of rigour, quality appraisal will be undertaken independently by two authors and then compared before agreement reached.

### Step 4: Data extraction

To ensure the most suitable information is extracted, a draft data extraction tool has been developed, based on RRR methodology ^15, 26^ (see *Extended data* ^21^). This tool will be pre-tested before use to ensure it captures all relevant information accurately. Data will be extracted from the article and checked by a second member of the research team. When extracting data, if an article does not include information relevant to a question in the form, the researcher will record this as ‘not reported’. The data extraction process will populate the data extraction table with evidence. Once the research team and sub-teams extract the data, the content from each team’s data extraction tables will be amalgamated into one single data extraction table to include all articles. 

### Step 5: Validation of findings

This RRR will be undertaken in consultation with a local reference panel. The local reference panel will consist of eight key intervention stakeholders’, and knowledge users such as healthcare professionals who will further develop and implement the interventions. RRR is grounded in the local context, with explicit, extensive, iterative engagement with a local reference group comprising representatives of potential knowledge users ^12^. The benefits of including key stakeholders and knowledge users in the process of a review include increased relevance, clarity and awareness of review findings ^27^. In addition to the local reference panel, an expert panel will consist of researchers from two universities in the east and one in the west of Ireland. An expert panel with the guidance of a librarian will help to identify the relevant articles that should be included in the review and contribute to tailoring the search strategy, synthesis of findings and verifying appropriate interpretation of results. This expert panel will consist of researchers and practitioners who have experience in RRR methodology pertinent to nursing and midwifery healthcare settings and also have a connection or previous collaboration with the three universities involved with this review. As this review will be conducted in the midst of a healthcare pandemic, all contact with stakeholders and knowledge users will be conducted virtually through secure online communication platforms. Initially, the local reference panel and an expert panel will define the research questions, review the inclusion/exclusion and search strings of the RRR to ensure clarity and consistency. Following title and full-text screen by the team of researchers, the expert panel will meet the research team to review selected articles to ensure all relevant articles are included. A final consensus meeting will be convened with the expert panel and local reference panel once synthesis of the literature is complete, to highlight the different enabling and constraining contextual factors and mechanisms which influence the implementation of QCP-Ms across seven practice areas.

### Step 6: Data synthesis

We will develop an approach to synthesis, including the following steps, as outlined by Rycroft-Malone *et al.* 2012 ^28^.

1. Organisation of extracted data into evidence tables

2. Theming by individual reviewers

3. Comparison of reviewers’ themes for a specific article and formulation of chains of inference from the identified themes

4. Linking of the chains of inference, and tracking and linking of articles

5. Hypotheses formulation (mechanism, context, outcome chains)

The thematic analysis framework ^29^ will be operationalised to analyse the findings from each selected paper within six non-linear planes; (1) familiarity with the information, (2) generate codes, search for themes, (3) review the themes, (4) define and (5) names the themes and (6) produce the findings. Selected papers will be imported into the software NVivo 23 ^30^. Results and discussion sections will be coded in order to identify context, mechanism, outcome configurations in the findings.

### Step 7: Refining the programme theory

The final stage involves the refining and testing of the programme theory, following synthesis of the data. It is envisaged that the programme theory will explain how and why QCP-M’s produce outcomes from changes in contexts and their resultant interactions with mechanisms. The local reference and expert panel will assist in refining the final theory by providing us with their expertise in the field. After completing all steps in this review, any stage may be revisited in order to ensure data saturation and ‘theory saturation’ ^21^. Once the review has been deemed to have reached data and theory saturation, no further documents will be added, and steps will not be repeated.

## Conclusions

Findings from this review will help to give insights into realism as a framework and how nursing and midwifery QCP-Ms have been implemented previously. Findings related to the barriers and facilitators to healthcare interventions/initiatives being evaluated could enable us to identify ways in which we can evaluate the bigger QCP-M’s project. This RRR will provide a nationally and internationally unique approach to measuring nurses and midwives’ contribution to quality and safe care, representing a departure from traditional methodologies and will provide a robust lens into phase two of the evaluation research project.

## Data availability

### Underlying data

No underlying data are associated with this article.

### Extended data

Figshare: Quality Care Metrics (QCP-Ms) in Nursing and Midwifery Care Processes: A Rapid Realist Review (RRR) Protocol, http://doi.org/10.6084/m9.figshare.13040333 ^21^.

This project contains the following extended data:

- PRISMA-P_Supp_A.docx (Supplementary Material A – PRISMA-P checklist)
- Supp_Mat_B.docx (Supplementary Material B - potential academic databases and grey literature databases)
- Supp_Mat_C.docx (Supplementary Material C – PRISMA flow diagram)
- Supplementary Material C (1) Data Extraction Tool.docx (Supplementary Material D – data extraction tool)

Data are available under the terms of the Creative Commons Zero "No rights reserved" data waiver (CC0 1.0 Public domain dedication).
