# Supplementary material for: Quality care process metrics (QCP-Ms) in nursing and midwifery care processes: a rapid realist review (RRR) protocol
Source: HRB Open Res. 2021 Jan 28;3:85. Originally published 2020 Nov 17. [Version 2] doi: 10.12688/hrbopenres.13120.2 (PMC7848854; doi:10.12688/hrbopenres.13120.2)
Supplement: Peer review history

# Peer review: Reviewer response for version 2

**DOI:** 10.21956/hrbopenres.14371.r28786

**Published:** 2021-01-29

**Authors:** Maura Dowling, Martina Giltenane

**Affiliations:** 1 School of Nursing and Midwifery, National University of Ireland, Galway, Ireland

The authors have provided comprehensive and clear responses to our suggestions and robust clarifications to our queries. Thank you. (There are just a few minor typos in the last paragraph of the introduction noted).

Is the study design appropriate for the research question?

Yes

Is the rationale for, and objectives of, the study clearly described?

Yes

Are sufficient details of the methods provided to allow replication by others?

Yes

Are the datasets clearly presented in a useable and accessible format?

Not applicable

Reviewer Expertise:

Maura Dowling: qualitative methodologies; qualitative evidence synthesis; concept analysis. Martina Giltenane: qualitative methodologies, modified Delphi, quality improvement and measurement.

We confirm that we have read this submission and believe that we have an appropriate level of expertise to confirm that it is of an acceptable scientific standard.

# Peer review: Reviewer response for version 1

**DOI:** 10.21956/hrbopenres.14229.r28476

**Published:** 2020-12-11

**Authors:** Mary Ellen Glasgow

**Affiliations:** 1 School of Nursing, Duquesne University, Pittsburgh, PA, USA

Well-written important article. I would suggest a bit more of a discussion/background of rapid realist review (RRR) and why it is an important methodology in these circumstances and nursing in particular. Rapid Realist Review’ methodology (RRR) has been developed as a tool for applying a realist approach to a knowledge synthesis process for quality improvement in time -sensitive scenarios or emerging clinical issues. The RRR methodology is designed to engage knowledge users and review stakeholders to define the research questions, and to streamline the review process. Results are typically presented with a focus on context-specific explanations for what works within a particular set of parameters rather than producing explanations that are potentially transferrable across all populations or contexts.

 Nursing can benefit from this methodology and I am glad to see the authors using this framework. .

Is the study design appropriate for the research question?

Yes

Is the rationale for, and objectives of, the study clearly described?

Yes

Are sufficient details of the methods provided to allow replication by others?

Yes

Are the datasets clearly presented in a useable and accessible format?

Yes

Reviewer Expertise:

Nursing education and leadership, quality improvement.

I confirm that I have read this submission and believe that I have an appropriate level of expertise to confirm that it is of an acceptable scientific standard.

# Response

**Published:** 2021-01-19

**Authors:** Marlize Barnard

**Affiliations:** University College Dublin, Ireland

**We truly appreciate this reviewer’s input and have amended the paper based on the valuable comments.**

**I would suggest a bit more of a discussion/background of rapid realist review (RRR) and why it is an important methodology in these circumstances and nursing in particular.**

 Underpinned by realist evaluation methodology (12) rapid realist review (RRR) has emerged as a popular approach in health service research and is well suited to a national evaluation of implementing QCP-Ms in Nursing and Midwifery practice. Unlike a systematic review which will control context, a realist review embraces contextual complexity making it a very appropriate approach for studying healthcare implementation and quality improvement (Davies *et al.,* 2019). It focuses on understanding how an intervention (i.e., QCP-Ms) works (or not) within a particular context, taking account of individual behaviours and system influences (15, 28). A RRR will identify the enabling and constraining dynamics influencing implementation. RRR review involves analysis and interpretation of existing data, in essence, it is the application of the realist approach to retrospective literature reviews (Pawson, 2002). RRR acknowledges that theories cannot and do not always offer explanations or predict outcomes in every context; for example, in patient safety programmes (Newton et al., 2011). However, RRR is an approach that suits situations where policymakers and practitioners require guidance to inform emergent decisions in practice. The ‘rapid’ review is deliberately intended to streamline evidence synthesis and provide practical outcome-based results in a short time frame.  Using an expert panel, it directly engages policy makers and knowledge users in the process which ensures the review remains relevant to the practice context (13, Davies et al., 2019). While this review directly informs QCP-Ms implementation in Nursing and Midwifery Practice in Ireland, it will also have transferrable learning for any other discipline or health system implementing similar improvement efforts.

# Peer review: Reviewer response for version 1

**DOI:** 10.21956/hrbopenres.14229.r28441

**Published:** 2020-12-02

**Authors:** Maura Dowling, Martina Giltenane

**Affiliations:** 1 School of Nursing and Midwifery, National University of Ireland, Galway, Ireland

This is a very relevant rapid realist review. It will illuminate the reasons for successful implementation of quality care metrics (QCMs) and the challenges and/or barriers to implementing QCMs in different contexts. This will guide healthcare providers in relation to measurement for improvement in the future to enable them to learn from processes used in healthcare settings within similar contexts.

**Introduction:**

 An explanation of the term ‘quality care-metrics’ would be useful.

 The authors identify that nurses and midwives are at the centre of care delivery, delivering high-quality and safe care, yet there is no definition describing what high-quality care entails. What is quality care? This explanation would strengthen the background.

 A specification of what indicators this paper will focus on would strengthen the paper i.e. all types of indicators structure, process and outcomes or just processes of care that are controlled by nurses and midwives. Within the protocol section process metrics are mentioned but an explanation of the difference between types of indicators coming earlier is recommended. 

 It is also recommended that the authors include an explanation that quality care metrics are made up of process indicators within the same clinical domain.

 Regarding outcomes, it is not clear if the authors hope to demonstrate that quality care metrics were effective and if any specific reported outcomes were to be reported?

**Research Question**

 Given the aim of the paper, we suggest that the authors consider additional research question(s), for instance: What are the barriers and/or challenges of implementing QCMs across Nursing and Midwifery Practice? 

**Methods**

Search strategy: The authors do not indicate if a librarian or information specialist is part of the team and if not part of the team will a librarian or information specialist assist with the search strategy?

Screening: The authors outline how discrepancies regarding article selection will be addressed but have not been explicit regarding how many authors (and who) will undertake title and abstract screening and full text screening is not outlined.

Quality appraisal: The authors outline that they will use either the CCAT or CASP and that ‘only papers above 25 [CCAT scores out of 40] will be accepted’. It would be more appropriate to use one quality appraisal tool across all studies and not exclude studies based on their score. Studies deemed lower quality may contribute useful findings to the review. Moreover, in terms of rigour, all quality appraisal should be undertaken independently by two authors and then compared before agreement reached.

**Some minor grammatical errors noted as follows:**

- Abstract-Background second last line QCM change to plural.
- Be consistent with the use of QCMs abbreviation or in full term. 
- Data Synthesis-step 6 grammar -(5) names [sic] the themes.

Is the study design appropriate for the research question?

Yes

Is the rationale for, and objectives of, the study clearly described?

Yes

Are sufficient details of the methods provided to allow replication by others?

Yes

Are the datasets clearly presented in a useable and accessible format?

Not applicable

Reviewer Expertise:

Maura Dowling: qualitative methodologies; qualitative evidence synthesis; concept analysis. Martina Giltenane: qualitative methodologies, modified Delphi, quality improvement and measurement.

We confirm that we have read this submission and believe that we have an appropriate level of expertise to confirm that it is of an acceptable scientific standard, however we have significant reservations, as outlined above.

# Response

**Published:** 2021-01-19

**Authors:** Marlize Barnard

**Affiliations:** University College Dublin, Ireland

**We would like to sincerely thank both reviewers for their extremely helpful input to this paper and we as a team have addressed all suggestions.**

****

**An explanation of the term ‘quality care-metrics’ would be useful.**

 Nursing metrics are agreed standards of measurement for nursing and midwifery care, where care can be monitored against agreed standards and benchmarks (Foulkes, 2011). According to HSE, QCP-Ms are sensitive to the influence of nurses and midwives appropriate for all care settings, aligned to evidence-based standards and agreed through national consensus. Nursing Quality Care Process Metrics provide the framework to identify gaps in care delivery, enabling action planning for quality improvement and provide a mechanism by which care providers can be accountable for the quality of their care delivery (HSE, 2018).

**The authors identify that nurses and midwives are at the center of care delivery, delivering high quality and safe care, yet there is no definition describing what high-quality care entails. This explanation would strengthen the background.**

 The WHO (2006), defined high quality of care as the extent to which health care services provided to individuals and patient populations improve desired health outcomes. In order to achieve this, health care must be safe, effective, timely, efficient, equitable and people-centred.

**A specification of what indicators this paper will focus on would strengthen the paper i.e. all types of indicators structure, process and outcomes or just processes of care that are controlled by nurses and midwives.**

 Quality measures are frequently classified into three types: structure, process and outcomes (Donabedian, 1988). Structure reflect factors such as the availability of staff and facilities, process considers whether care interventions adhered to best practice guidance, and outcomes consider the changes because of care delivered.

**Within the protocol section process metrics are mentioned but an explanation of the difference between types of indicators coming earlier is recommended.**

 We have removed the term indicator as the RRR is focused on the quality care process metrics only.

**It is also recommended that the authors include an explanation that quality care metrics are made up of process indicators within the same clinical domain.**

 Quality measures are frequently classified into three types: structure, process and outcomes (Donabedian, 1988). Structure reflect factors such as the availability of staff and facilities, process considers whether care interventions adhered to best practice guidance, and outcomes consider the changes because of care delivered.

**Regarding outcomes, it is not clear if the authors hope to demonstrate that quality care metrics were effective and if any specific reported outcomes were to be reported?**

 We have stated that we are testing the CMO’s of reported studies to show evidence of effectiveness / non-effectiveness, reasons and outcomes whether positive or negative-see below.

 By testing context-mechanisms-outcomes (CMO) configurations, this will enable us to understand how, why, for whom, and in what contexts the intervention, i.e., QCP-M’s are more or less likely to work and how it produces its desired and undesired outcomes ^14,15, ^.

**Research Question**

**Given the aim of the paper, we suggest that the authors consider additional research question(s), for instance: What are the barriers and/or challenges of implementing QCMs across Nursing and Midwifery Practice?**

**Research Question:**

 What factors enable the successful implementation of a suite of quality care process nursing/midwifery metrics across all areas in nursing and midwifery practice? 

 Additional sub research questions

- In nursing and midwifery quality care process metrics, what contexts and mechanisms lead to positive implementation outcomes?
- In nursing and midwifery quality care process metrics, what contexts and mechanisms lead to negative implementation outcomes?
- What were the dominant outcome patterns in identified contexts?

**Methods**

**Search strategy: The authors do not indicate if a librarian or information specialist is part of the team and if not part of the team will a librarian or information specialist assist with the search strategy?**

 An expert panel with the guidance of a librarian will help……

**Screening: The authors outline how discrepancies regarding article selection will be addressed but have not been explicit regarding how many authors (and who) will undertake title and abstract screening and full text screening is not outlined.**

 Following this, the titles and abstracts will be screened by two teams of two reviewers independently. Similarly full text will be screened by two teams of two reviewers independently.

**Quality appraisal: The authors outline that they will use either the CCAT or CASP and that ‘only papers above 25 [CCAT scores out of 40] will be accepted’. It would be more appropriate to use one quality appraisal tool across all studies and not exclude studies based on their score. Studies deemed lower quality may contribute useful findings to the review. Moreover, in terms of rigour, all quality appraisal should be undertaken independently by two authors and then compared before agreement reached.**

 The CCAT critical appraisal tool will be utilised to ensure the highest quality papers and grey literature will be included in the review. More importantly, the CCAT is a guide to become more objective in assessing papers (personal correspondence Crowe 2020). Moreover, in terms of rigour, quality appraisal will be undertaken independently by two authors and then compared before agreement reached.
